# The mechanical, electronic, optical and thermoelectric properties of two-dimensional honeycomb-like of XSb (X = Si, Ge, Sn) monolayers: a first-principles calculations†

**DOI:** 10.1039/d0ra05587e

**Published:** 2020-08-17

**Authors:** Asadollah Bafekry, Fazel Shojai, Doh M. Hoat, Masoud Shahrokhi, Mitra Ghergherehchi, C. Nguyen

**Affiliations:** Department of Physics, University of Guilan 41335-1914 Rasht Iran Bafekry.asad@gmail.com; Department of Physics, University of Antwerp Groenenborgerlaan 171 B-2020 Antwerp Belgium; Department of chemistry, Faculty of sciences, Persian gulf university Bushehr 75169 Iran; Computational Laboratory for Advanced Materials and Structures, Advanced Institute of Materials Science, Ton Duc Thang University Ho Chi Minh City Vietnam; Faculty of Applied Sciences, Ton Duc Thang University Ho Chi Minh City Vietnam; Department of Physics, Faculty of Science, University of Kurdistan 66177-15175 Sanandaj Iran; College of Electronic and Electrical Engineering, Sungkyunkwan University Suwon Korea mitragh@skku.edu; Institute of Research and Development, Duy Tan University Da Nang 550000 Vietnam

## Abstract

Herein, by using first-principles calculations, we demonstrate a two-dimensional (2D) of XSb (X = Si, Ge, and Sn) monolayers that have a honey-like crystal structure. The structural, mechanical, electronic, thermoelectric efficiency, and optical properties of XSb monolayers are studied. *Ab initio* molecular dynamic simulations and phonon dispersion calculations suggests their good thermal and dynamical stabilities. The mechanical properties of XSb monolayers shows that the monolayers are considerably softer than graphene, and their in-plane stiffness decreases from SiSb to SnSb. Our results shows that the single layers of SiSb, GeSb and SnSb are semiconductor with band gap of 1.48, 0.77 and 0.73 eV, respectively. The optical analysis illustrate that the first absorption peaks of the SiSb, GeSb and SnSb monolayers along the in-plane polarization are located in visible range of light which may serve as a promising candidate to design advanced optoelectronic devices. Thermoelectric properties of the XSb monolayers, including Seebeck coefficient, electrical conductivity, electronic thermal conductivity, power factor and figure of merit are calculated as a function of doping level at temperatures of 300 K and 800 K. Between the studied two-dimensional materials (2DM), SiSb single layer may be the most promising candidate for application in the thermoelectric generators.

## Abstract 2

Herein, by using first-principles calculations, we demonstrate a two-dimensional (2D) of XSb (X = Si, Ge, and Sn) monolayers that have a honey-like crystal structure.

## Graphical abstract

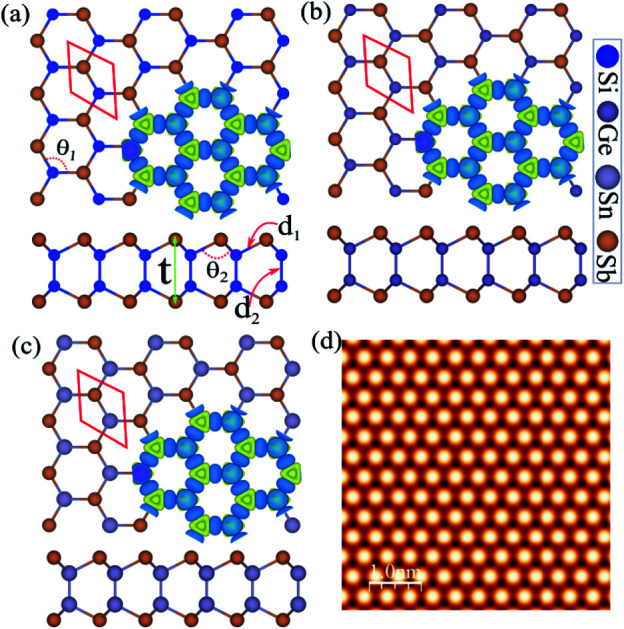

## 1 Introduction

Since the first synthesis of graphene in 2004,^1^ considerable research efforts have been devoted to the synthesis of other members of the big family of 2DM. This family of materials exhibits a broad range of unique electronic, optical, thermal, and mechanical properties, therefore making them promising candidates for many technological and scientific applications.^2–5^ However, graphene and a few other 2D elemental materials like silicene, phosphorene, and borophene as well as binary MoS_2_ have been the main focus of research in nanomaterials science. Just recently, layered group IVA–VA binary compounds with several experimentally observed stoichiometries (IVV (GeP, GeAs, SiP, SiAs),^6,7^ IVV2, (SiP_2_, SiAs_2_, GeAs_2_)^8,9^ IVV3 (GeP_3_, SnP_3_),^10^ and IVV5 (GeP5),^11^) have also received an increasing research attention.

The electronic structure of these 2D binary compounds are expected to be different than that of each of their 2D elemental parents (group IV and V). Group IV 2DM are all semiconductors with band gaps in the range 0.67–2.5 eV.^12,13^ Despite of several merits of group-V 2DM, they lack of air stability which hinders their device applications toward sustainable competitors.^14^ An allotrope of layered bulked antimonene with a phosphorene-like puckered structure is predicted to exhibit ferroelectric properties with Curie temperature above room temperature.^15^ Group IV 2DM beyond graphene, including silicene, germanene, and stannane are among the first fabricated elemental 2DM.^16–18^ Calculations predict that if spin-orbital coupling (SOC) is not taken into account, their monolayers with buckled honeycomb lattices are zero gap semiconductors with Dirac cones similar to that in graphene monolayer. It is found that the inclusion of SOC opens small band gaps of 1.5, 24, and 100 meV at the Dirac point for silicene, germanene, and stanene.^19^ Most interestingly, an ultraflat stanene monolayer with room temperature topological properties was discovered in a recent experiment.^18^ Their electronic structure can be also modulated by the presence of different substrates and types stacking patterns.^20^ Because of different normal coordination numbers of elements of group-IV and V, their combination with various stoichiometries expectedly results in different crystal lattices with distinct bonding patterns compared to those of their elemental parents, and consequently different electronic properties.

The ease of exfoliation,^21–26^ mechanical properties,^27^ band gap,^28,29^ electrical transport,^23,24,30^ thermal conductivity,^28^ thermoelectric efficiency^28^ and photocatalytic activity^29,31^ of group IV–V 2DM have been the subject of several theoretical and experimental studies. Interestingly, it has been also theoretically shown that stoichiometries of IVV, IVV2, and IVV3 exhibit intriguing polymorphic natures in two dimension. As an example, a hexagonal GaS-like lattice (V–IV–IV–V) with *P*6*m*2 space group has been proposed for IVV compounds, in addition to their experimentally observed layered monoclinic phase.^32–36^ Calculations shows that GaS-like IVV monolayers are all semiconductors except for CBi and PbN, which exhibits metallic behavior.^32^ It has been shown that their electronic structure can be effectively tuned by applying different types of strain, electric field, and forming heterojunction with other 2DM like graphene.^36–38^ Among these materials, other two dimensional nanomaterials have attracted intense attention during the past few years.^39–62^

Here, motivated by: on the one hand, the recent experimental realization of 2D IVV monolayers with novel properties,^63,64^ and on the other hand, by distinct electronic properties of polymorphs of different 2DM, we have examined the structural, mechanical, electronic, thermoelectric efficiency, and optical properties of XSb (X = Si, Ge, and Sn) monolayers by using density functional theory. Our results provide qualitative and quantitative information on the importance of the chemical composition and structural configuration of XSb (X = Si, Ge, and Sn) monolayers and guide experimental studies for next generation applications.

## 2 Method

In this work, we report results of our DFT calculations for the electronic structure as implemented in the OpenMX 3.8 package.^65^ This code determines the eigenfunctions and eigenvalues of the Kohn–Sham equations self-consistently using norm-conserving pseudopotentials.^66^ The Perdew–Burke–Ernzerhof approach from the generalized gradient approximation (PBE-GGA)^67^ is applied to describe the exchange–correlation functional. The wave functions are obtained from the linear combination of multiple pseudoatomic orbitals (LCPAOs), which can be generated by a confinement scheme.^68,69^ After convergence tests, we chose energy cutoff 400 Ry for pristine monolayers. The atomic positions are optimized using a quasi-Newton algorithm for atomic force relaxation, where the structure was fully relaxed until the force acting on each atom was less than 1 meV Å^−1^. The Monkhorst–Pack scheme^70^*k*-point sampling was chosen to be 23 × 23 × 1 for primitive unit cell and scaled according to the size of the supercell. The XSb monolayers were modeled as a periodic slab with a sufficiently large vacuum layer (20 Å) in order to avoid interaction between adjacent layers. To get a clear picture in the view of van der Waals interactions, dominated in layered XSb monolayers, we used the empirical dispersion method of DFT-D2.^71^ The vibrational characters of XSb monolayers are obtained by performing the finite-displacement method within PHONOPY code.^72^ Furthermore, we also provide the scanning tunneling microscopy (STM) simulations using the Tersoff–Hamann^73^ in WSxM package.^74^

The optical calculations, such as real and imaginary parts of dielectric tensor, absorption coefficient and reflectivity were performed in the random phase approximation (RPA)^75^ method constructed over the screened hybrid Heyd–Scuseria–Ernzerhof functional (HSE06).^76^ The wave function in the interstitial region were expanded in terms of plane waves with a cut-off parameter of *R*_MT_*K*_max_ = 8.5, where *R*_MT_ denotes the smallest atomic sphere radius and *K*_max_ expresses the largest *k* vector in the plane-wave expansion. The optical properties were evaluated using a dense *k*-point grid of 18 × 18 × 1 *Γ*-centered Monkhorst–Pack and setting Lorentzian broadening with gamma equal to 0.05 eV. For more details about calculations of optical properties see ESI.† Thermoelectric properties of the XSb monolayers have been calculated by means of the interpolation of the electronic band structure supported by the BoltzTraP code,^77^ in which the semiclassical Boltzmann transport theory is implemented. It is important to mention that the accuracy is extremely sensitive to the band structure, therefore, we use a very dense *k*-mesh of 36 × 36 × 1 during thermoelectric properties calculations.

## 3 Structural properties

**Fig. 1:**
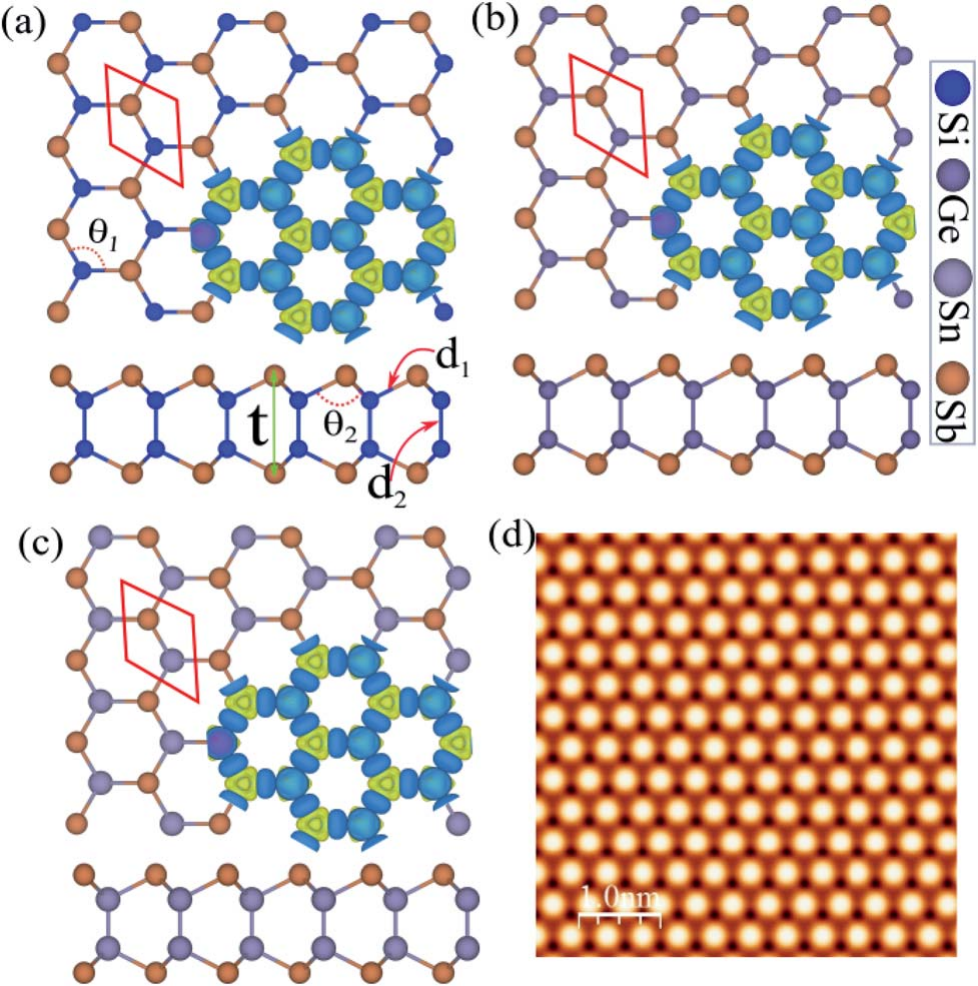
Atomic structures of (a) SiSb, (b) GeSb and (c) Sn monolayers with the primitive unit cell indicated by a red hexagonal. Difference charge density indicated in the same panel. The blue and yellow regions represent the charge accumulation and depletion, respectively. (d) Simulated STM images of SiSb monolayer. The inset structure represents repeating the unit cell.

The atomic structure was used to construct three new binary monolayers, with a three fold-coordinated X (Si, Ge and Sn) and Sb atoms in a hexagonal unit cell containing four atoms, as shown in Fig. 1(a). Notice that the crystal structures of XSb (X = Si, Ge, Sn) monolayers consists of 2-X layers sandwiched between Sb-layers in the Sb–X–X–Sb order. The lattice constants of SiSb, GeSb and SnSb monolayers are calculated to be 3.97, 4.04 and 4.35 Å, respectively. The bond lengths of *d*_1_ are found to be 2.60 Å (Si–Sb), 2.65 Å (Si–Sb) and 2.83 Å (Si–Sb), while the *d*_2_ are determined 2.32 Å (Si–Si), 2.45 Å (Ge–Ge) and 2.81 Å (Sn–Sn). The structural and electronic parameters of the XSb (X = Si, Ge, Sn) monolayers are listed in Table 1.

**Table 1:** The structural, electronic and magnetic parameters of XSb (X = Si, Ge, Sn) monolayers shown in Fig. 1(c). The corresponding structural and electronic parameters including lattice constant *a*; the bond length between X–Sb atoms *d*_1_ and X–X atoms *d*_1_, where X = Si, Ge and Sn; the bond angles between Sb–X–Sb atoms *θ*_1_ and Sb–X–X *θ*_2_ the thickness defined by the distance between the largest and smallest *z* coordinates of Sb atoms (Δ*z*); the cohesive energy per atom, (*E*_coh_); the charge transfer (Δ*Q*) between atoms; the band gap within PBE (*E*^PBE^_g_), band gap within HSE (*E*^HSE^_g_), the band gap inside parentheses is with considering SOC and is given in eV. The VBM and CBM positions. In-plane stiffness (*C*) and Poisson's ratio (*ν*)

	*a* (Å)	*d*_1_ (Å)	*d*_2_ (Å)	Δ*z* (Å)	*θ*_1_ (°)	*θ*_2_ (°)	*E*_coh_ (eV pe atom)	Δ*Q* (e)	*E* ^PBE^_g_ (eV)	*E* ^HSE^_g_ (eV)	VBM/CBM	*C* (N m^−1^)	*ν*
SiSb	3.97	2.60	2.32	4.78	99.49	118.20	−3.45	0.09	1.43 (1.23)	1.68 (1.48)	*Γ*/*M*	88.30	0.25
GeSb	4.04	2.65	2.45	4.99	99.06	118.54	−3.34	0.30	0.71 (0.51)	0.99 (0.77)	*Γ*/*M*	67.77	0.31
SnSb	4.35	2.83	2.81	5.16	97.69	119.61	−3.21	0.05	0.77 (0.57)	1.00 (0.73)	*Γ*/*M*	58.01	0.32

The difference charge density is shown in Fig. 1(a) in the same panel, where the blue and yellow regions represent the charge accumulation and depletion, respectively. Notice that the negatively charged Sb atoms are surrounded by positively charged X (Si, Ge and Sn) atoms, which indicates a charge transfer from X atoms to Sb atom. The difference charge density (Δ*ρ*) is defined as:

1 Δ*ρ* = *ρ*_tot_ − *ρ*_X_ − *ρ*_Sb_

where *ρ*_tot_, *ρ*_X_ and *ρ*_Bi_ represents the charge densities of the XBi and isolated atoms, respectively. Notice that each Sb atom gains about 0.09, 0.30 and 0.05*e* from the adjacent Si (in SiSb), Ge (in GeSb) and Sn (in SnSb), respectively. The charge redistribution is due to the different electro-negativities of Si (1.9) (Si), Ge (2), Sn (1.96) and Sb (2.05).

To calculate the cohesive energy *E*_coh_, we use the standard expression:2
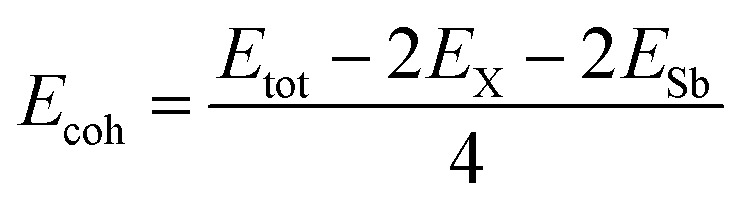
where *E*_X_ and *E*_Sb_ represent the energies of isolated single X (Si, Ge and Sn) and Sb atoms, respectively; *E*_tot_ represents the total energy of the XSb monolayer. The cohesive energy of SiSb, GeSb and SnSb, are found to be −3.45, −3.34 and −3.21 eV per atom, respectively. The more negative values for cohesive energies suggest more structural stability of the studied monolayers and the structures represent more stability when the atoms are lighter.

**Fig. 2:**
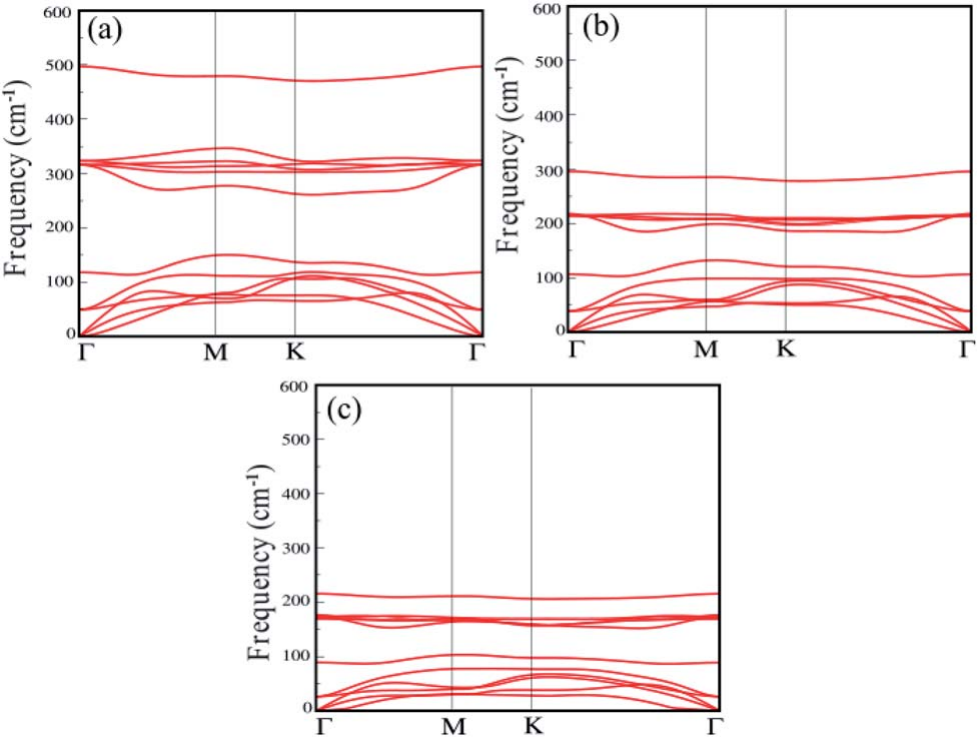
Phonon dispersion spectra of the (a) SiSb, (b) GeSb and (c) SnSb monolayers.

The dynamical stability of each single-layer of group IV–V compounds are examined in terms of their phonon band dispersions. Our results reveal that all the single-layers are dynamically stable with all phonon branches having fully real frequencies. The results of phonon dispersion along the high symmetry points in the BZ are shown in the Fig. 2(a)–(c). We find that the phonon dispersion is completely positive, and the minimum of the acoustic branch is linear around the *Γ* point, which demonstrates that XSb monolayer is kinetically stable. In addition, the thermal stability are examined by performing *ab initio* molecular dynamics (AIMD) simulations using NVE ensemble. For the AIMD simulations, 32-atom supercell is used for each monolayer with a *k*-mesh of 5 × 5 × 1 as shown in Fig. S1 of the (ESI†). We find that the free energy curves as a function of time-step for XSb monolayers fluctuate around the equilibrium positions, and their crystal structures corresponding to the last free energy maximum in the *T* = 300 K case, which are shown on the right part of Fig. S1(a)–(c),† still show no significant structural differences as compared with their initial crystal structures. This means that these materials can be stable at room temperature. Therefore, our calculations mentioned above provide an authentic test for the stability of XSb monolayer.

## 4 Mechanical properties

We also examined the mechanical properties of XSb monolayers by calculating the strain energy in the framework of harmonic approximation. The strain energy caused by caused by different types of uniaxial (*x* and *y* directions) and biaxial strains, is supposed to be always positive for a carefully relaxed 2DM and it is defined as:

3 *E*_s_ = 1/2*C*_11_*E*_*xx*_^2^ + 1/2*C*_22_*E*_*yy*_^2^ + *C*_12_*E*_*xx*_*E*_*yy*_ + 2*C*_66_*E*_*xy*_

where the *E*_s_, *E*_*xx*_, *E*_*yy*_ and *E*_*xy*_ are strain energy per unit area, uniaxial strains along the *X* and *Y* axes, and shear strain along the *XY* direction, respectively. *C*_11_, *C*_22_, *C*_12_ and *C*_66_ are linear elastic constants, and they can be simply calculated using a series of parabolic fitting of *E*_s_ as function of uniaxial and biaxial strains. *C*_11_ and *C*_22_ are equal for XSb monolayers because of their hexagonal crystal symmetry. The calculated elastic constants are found to be *C*_11_ = *C*_22_ = 94.09 N m^−1^, *C*_12_ = 23.33 N m^−1^, and *C*_66_ = 8.49 N m^−1^ for SiSb monolayer. Notice that *C*_11_ = *C*_22_ = 74.77 N m^−1^, *C*_12_ = 22.88 N m^−1^, and *C*_66_ = 15.09 N m^−1^ for GeSb, and *C*_11_ = *C*_22_ = 64.58 N m^−1^, *C*_12_ = 20.6 N m^−1^, and *C*_66_ = 12.54 N m^−1^ for SnSb monolayer. The mechanical properties of a hexagonal 2DM are described by two independent parameters of in-plane stiffness (*C*) and Poisson's ratio (*n*). The in-plane stiffness along *X* and *Y* directions are equal for XSb monolayers, and they obtained from the elastic constants as follows: 
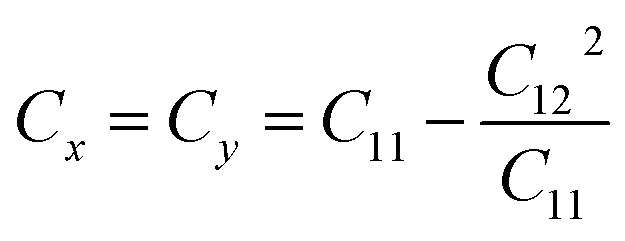
. The stiffness values are estimated to be 88.30 N m^−1^ for SiSb, 67.77 N m^−1^ for GeSb, and 58.01 N m^−1^ for SnSb monolayers. As it can be seen the in-plane hardness of these materials expectedly decreases from SiSb to SnSb. The Poisson's ratio along *X* and *Y* direction are also equals along *X* and *Y* direction for each monolayer, and they are calculated using 
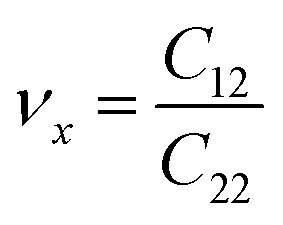
, 
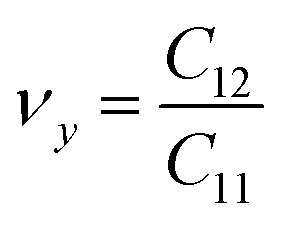
. The calculated Poisson's ratios are found to be *ν*_*x*_ = *ν*_*y*_ = 0.25 for SiSb, *ν*_*x*_ = *ν*_*y*_ = 0.31 for GeSb, *ν*_*x*_ = *ν*_*y*_ = 0.32 for SnSb monolayers.

## 5 Electronic properties

**Fig. 3:**
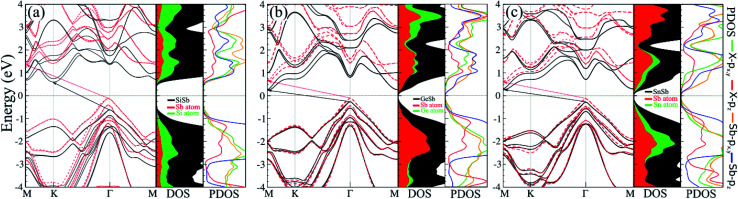
(a) Electronic band structure, with corresponding DOS and PDOS of (a) SiSb, (b) GeSb and (c) SnSb monolayers. The band structure within PBE and HSE with considering SOC indicated by black line and red dashed-line, respectively.

The electronic band structure, with corresponding DOS and PDOS of XSb monolayers are shown in Fig. 3(a)–(c). Our results that the XSb monolayers are a semiconductor, with indirect band gap of 1.43 (SiSb), 0.71 (GeSb), 0.77 (SnSb) eV, are obtained within PBE functional without SOC. In addition, the valance band maximum (VBM) and conduction band minimums (CBM) of SiSb are located at the *Γ* and *K* points, respectively. While VBM and CBM for the GeSb and SnSb are located at the *Γ* and *M* points, respectively. Notice that with considering of SOC, the band gaps of SiSb, GeSb and SnSb decrease to 1.23, 0.51 and 0.57 eV, respectively. Since the XSb monolayers are semiconductor, the HSE06 functional was also used to evaluate the electronic band structure as depicted in Fig. 3(a)–(c) with red-dashed line. Based on the acquired band structure by HSE06 method, the indirect band-gaps of SiSb, GeSb and SnSb monolayers were estimated to be 1.68, 0.99 and 1 eV, while their when SOC considered within HSE, indirect band-gaps decrease to 1.48 (SiSb), 0.77 (GeSb), 0.73 eV (SnSb). In order to understand the contribution of different orbitals to the electronic states and the bonding characteristics of XSb monolayers, we carry out the calculations of density of state (DOS) and partial DOS (PDOS) as shown in Fig. 3(a)–(c). It is observed that the states near the Fermi-level have contributions from p orbitals of X and Sb. The contributions from the p_*z*_ orbitals of X and Sb are much higher than that from p_*x*,*y*_-orbitals. The fact that the p_*z*_-orbitals are dominant is caused by the sp^3^-like bond of X and the sp^2^-like bond of Sb forming the XSb monolayers.

Our results also showed the electron effective mass is 0.241, 0.204 and 0.289
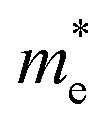
 for GeSb, SiSb and SnSb monolayers, respectively. The effective hole masses were obtained to be 0.102 (439), 112 (450) and 105 (461)
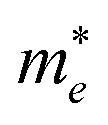
 for GeSb, SiSb and SnSb monolayers. Note that there are two bands as valance band maximum (VBM) in the band structure hence we reported the effective hole masses for both bands. The greater values in the parenthesis are the hole effective masses for the less curved bands. These light electron and hole effective masses lead to the high carriers mobility in this nanostructure.

## 6 Optical properties

**Fig. 4:**
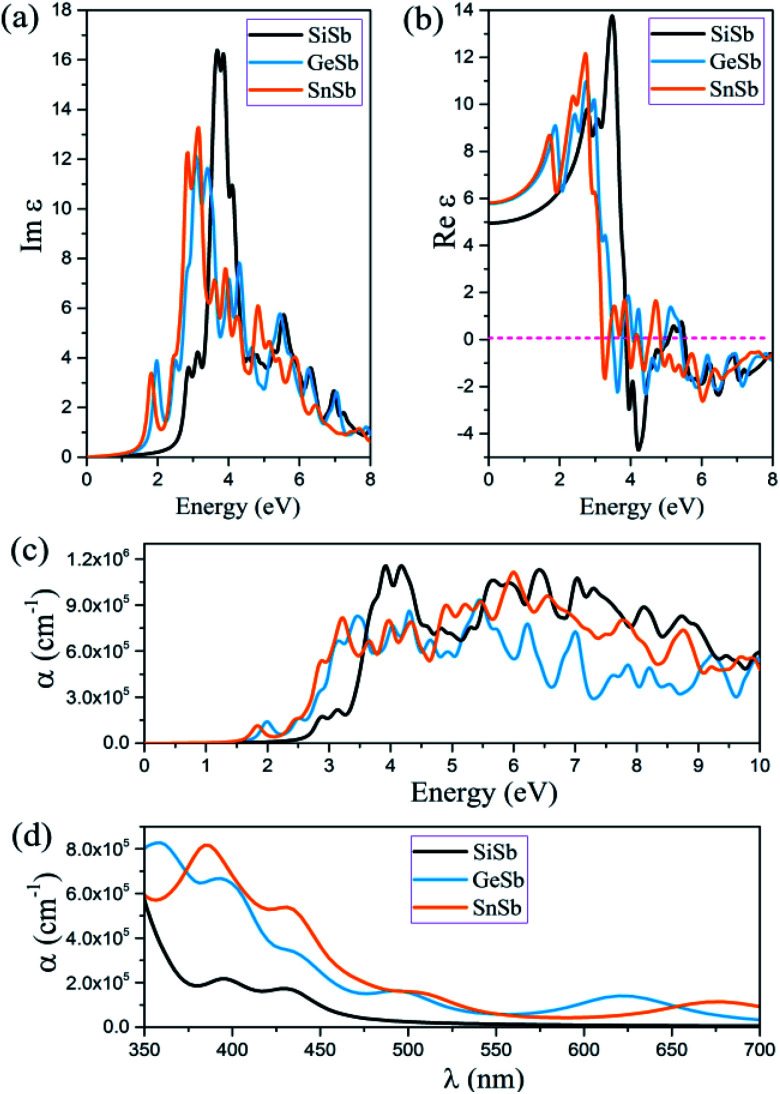
(a) Imaginary and (b) real parts of the dielectric function as a function of photon energy, (c) optical absorption spectra as a function of photon energy of the SiSb, GeSb and SnSb monolayers for the in-plane polarization, predicted using the RPA + HSE06 approach. (d) A comparison of optical absorption spectra as a function of wavelength, for aforementioned nanosheets in the visible range (350–700 nm) of light.

In this section applying the RPA method constructed over HSE06, the optical properties of novel 2D XSb (X is Si, Ge and Sn) monolayers, such as the imaginary and real part of dielectric function and absorption coefficient are calculated. Because of the strong depolarization effect in the 2D planar geometry for light polarization perpendicular to the plane, only the optical properties for light polarization parallel to the plane are reported. It is worth noting that all these structures have symmetric geometry along the *x*- and *y*-axes. The imaginary and real parts of the dielectric function of these 2D systems for the in-plane polarized directions were calculated and the acquired results are illustrated in Fig. 4. The first peak of Im(*ε*_*αβ*_) occurs at 2.89, 2.01 and 1.83 eV for the SiSb, GeSb and SnSb monolayers, respectively. These results indicate that the first peaks of Im(*ε*_*αβ*_) for all monolayers are in visible range of light along the in-plane polarizations and are related to π → π* transitions. The main peak of Im(*ε*_*αβ*_) was observed at energy of 3.70, 3.05 and 3.10 eV for the SiSb, GeSb and SnSb monolayers (see Fig. 4(a)). The static dielectric constants (real part of the dielectric constant at zero energy) were calculated to be 4.09, 5.78 and 5.80 for the SiSb, GeSb and SnSb monolayers, respectively (see Fig. 4(b)). The absorption coefficient *α* for all studied systems along in-plane polarization is plotted in Fig. 4(c). The first absorption peaks for the SiSb, GeSb and SnSb monolayers occur at energy of 2.86, 1.99 and 1.85 eV, respectively, which are in the visible range of light. This renders their potential applications in optoelectronic devices in the visible spectral range. From the results shown in the Fig. 4(d), it can be concluded that the absorption coefficient for the GeSb monolayer in the wavelength range of 350–375 and 550–650 nm is larger than those of SiSb and SnSb systems while in the wavelength range of 375–550 and 650–700 nm the absorption coefficient of SnSb is the largest. In general, the high absorption coefficients were attained (∼105 cm^−1^) for all these novel 2D systems in visible range of light which may be interesting for visible-light optoelectronic applications.

## 7 Thermoelectric properties

Thermoelectric properties of the XSb monolayers have been calculated using the semiclassical Boltzmann transport theory within framework of the rigid band approximation and constant scattering time approximation. As a first step, the transport distribution tensors *σ*_*αβ*_(*ϵ*) is calculated *via* interpolation of the electronic band structure by the following expression:4
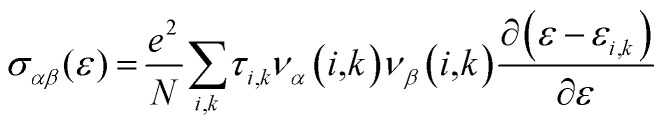
herein, *ν*(*i*,*k*) is the group velocity component with tensor indices *α* and *β*; *N* refers to the *k*-point number and *τ* denotes relaxation time. Then the Seebeck coefficient, electrical conductivity and electronic thermal conductivity are deduced from *σ*_*αβ*_(*ϵ*) as follow:5
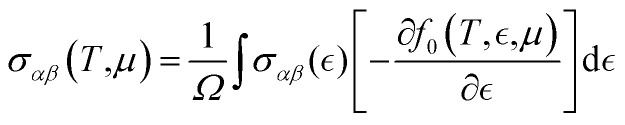
6
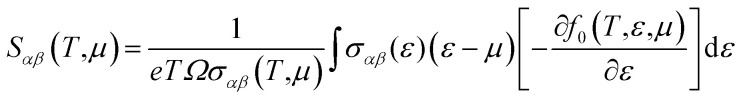
7
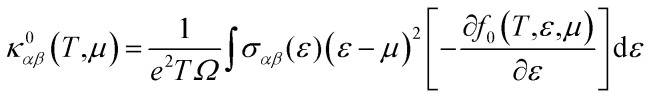


**Fig. 5:**
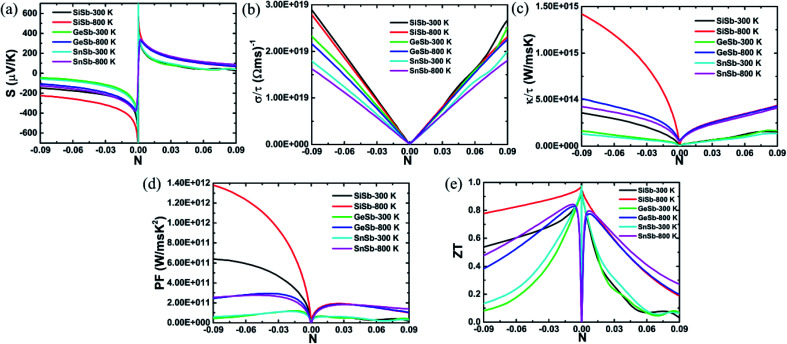
(a) Seebeck coefficient, (b) electrical conductivity, (c) thermal electronic conductivity, (d) power factor and (e) figure of merit of XSb monolayers at 300 and 800 K.

The parameters including Seebeck coefficient, electrical conductivity, thermal electronic conductivity, power factor and figure of merit are examined as a function of doping level *N* at temperatures of 300 and 800 K. Results are given in Fig. 5, in which negative(positive) values of *N* correspond to the electron(hole) concentration.

Fig. 5(a) shows the Seebeck coefficient (thermopower) behavior of the XSb monolayers depending on the charge carrier concentration. This parameter represents the potential induced by temperature difference cross material. Doubtless that high potential in material to push the charge carriers from high temperature to low temperature zones is expected. Note that this parameter takes large values at low concentration of both electron and hole. According to increase the doping level, the thermopower shows a decreasing trend. The reduction rate is considerably large for carrier concentration up to ±0.3, beyond of these values the decrease becomes slower. Results indicate that large thermoelectric potential may be achieved by small materials doping and *vice versa*. In the case of n-doping, the SiSb single layer Seebeck coefficient is larger than those of the GeSb and SnSb monolayers, where no important difference of these last is noted. When the materials are p-doped, the thermopower of all three monolayers is quite similar. In all cases, the increase of temperature may be favorable for the thermoelectric potential inducing a significant increase of this important thermoelectric properties.

In Fig. 5(b), the electrical conductivity of the XSb monolayers is plotted as a function of charge carrier concentration. Thermoelectric materials are expected to exhibit large electrical conductivity, that is, facilitating the formation of the charge carriers flow. It can be seen that the electrical conductivity increases nearly linearly with the doping level of both electron and hole, showing a contrary carrier-dependence in comparison with the Seebeck coefficient. This parameter decreases in the following order: SiSb → GeSb → SnSb. At given doping level, increasing temperature will induce a reduction of the electrical conductivity of studied 2DM, con exception of the p-doped SiSb single layer for whose electrical conductivity the temperature increase from 300 to 800 K may not influence significantly.

The XSb monolayers thermal electronic conductivity as a function of charge carrier concentration is illustrated in Fig. 5(c). Low values of the thermal electronic conductivity are desirable for the thermoelectric performance of materials. However, the thermal electronic conductivity and electrical conductivity may show proportionality at given temperature as theoretically established by the Wiedemann–Franz law: *κ*_el_ = *σLT*, here *L* is Lorenz number and *T* is absolute temperature. From the figure, one can see that at fixed temperature the thermal electronic conductivity increases with increasing the doping level, satisfying the Wiedemann–Franz law. However, this parameter exhibits an important increasing trend when the temperature is raised from 300 to 800 K. In the case of n-doping, the electronic thermal conductivity of the monolayers decreases in the X atom order: Si → Ge → Sn. While they possess a quite similar thermal electronic conductivity in the case of p-doping.

Power factor is a key thermoelectric parameter that links the Seebeck coefficient and electrical conductivity by the following formula: PF = *S*^2^*σ*. This parameter characterizes the electricity production of materials. Plots of the XSb monolayers power factor as a function of charge carrier concentration are given in Fig. 5(d). At small doping level, the power factor is extremely small, which is due to the negligible electrical conductivity values. According to increase the charge carrier concentration, this parameter increases rapidly to achieve its maximum values, and then it shows decreasing trend, unless the n-doped SiSb single layer whose power factor continuous increasing beyonds of the considered concentration range, the behavior may be attributed to its large values of both Seebeck coefficient and electrical conductivity. In the case of adding electron doping, the power factor decreases in the order SiSb → GeSb → SnSb. While when adding holes to the systems, no remarkable difference is noted. Increasing temperature may be a good approach to reach a high power factor value of the 2DM studied here. Results suggest that the electric conductivity may possess the dominant role on producing electricity over the thermopower.

Figure of merit measures the thermoelectric efficiency of materials, which is determined by the following formula: 
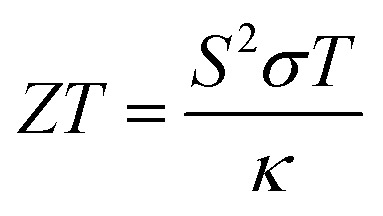
. Large figure of merit values suggest good thermoelectric performance of materials. Fig. 5(e) displays the plots of XSb monolayers figure of merit as a function of charge carrier concentration. At room temperature, this parameter can reach values close to unity at an extremely small doping level, then it decreases with the carrier concentration. It can be noted that in the case of n-doping, the decreasing rate increases in the following order SiSb → SnSb → GeSb. Unlikely, at 800 K, according to increase the doping level the figure of merit firstly increases to reach its maximum of 0.968(0.940) of SiSb, 0.828(0.776) of GeSb and 0.843(0.797) of SnSb at doping level of −0.0038(0.0004), −0.0077(0.0072) and −0.0082(0.0070), respectively. A higher doping level will induce a reduction of this parameter. It seems that the electron doping may be better approach than hole doping for a good thermoelectric performance of the studied 2D single layers because of larger figure of merit values at given doping level and temperature. It is worth mentioning that the figure of merit reduction when increasing the doping levels suggest the important (and negative) role of the electronic thermal conductivity. Therefore, in order to obtain a better thermoelectric performance of the studied 2DM, it is expected to reduce the thermal conductivity, which can be achieved by introducing the scattering centers.

## 8 Conclusion

In summary, we have studied the optoelectronic, mechanical, and thermoelectric properties of single layers of XSb (X = Si, Ge, and Sn) by using first-principles calculations. The phonon dispersion and *ab initio* molecular dynamic simulations calculations suggests theirs good thermal and dynamical stabilities. We found that the SiSb, GeSb and SnSb monolayers are semiconductor with band gap of 1.48, 0.77 and 0.73 eV, respectively, with inclusion of SOC effect. Our analysis of optical properties of studied monolayers indicates that the first absorption peaks of these novel 2DM along in-plane polarizations are located in visible range of light. The high absorption coefficients were attained for all these novel 2D systems in visible range of light may be desirable for employment in optoelectronic nanodevices. Calculated thermoelectric properties suggest the good performance of the materials studied here due to their large figure of merit values, which are close to unity. However, SiSb monolayer exhibits the most promising thermoelectric properties as at the same charge carrier concentration, it possesses large figure of merit values. Moreover, the thermoelectric performance may be enhanced considerably by increasing temperature from 300 to 800 K.

## Conflicts of interest

The authors declare that there are no conflicts of interest regarding the publication of this paper.

## Supplementary Material

RA-010-D0RA05587E-s001
