# Correction: The mechanical, electronic, optical and thermoelectric properties of two-dimensional honeycomb-like of XSb (X = Si, Ge, Sn) monolayers: a first-principles calculations

**DOI:** 10.1039/d0ra90090g

**Published:** 2020-08-26

**Authors:** Asadollah Bafekry, Fazel Shojaei, Doh M. Hoat, Masoud Shahrokhi, Mitra Ghergherehchi, C. Nguyen

**Affiliations:** Department of Physics, University of Guilan 41335-1914 Rasht Iran Bafekry.asad@gmail.com; Department of Physics, University of Antwerp Groenenborgerlaan 171 B-2020 Antwerp Belgium; Department of Chemistry, Faculty of Sciences, Persian Gulf University Bushehr 75169 Iran; Computational Laboratory for Advanced Materials and Structures, Advanced Institute of Materials Science, Ton Duc Thang University Ho Chi Minh City Vietnam; Faculty of Applied Sciences, Ton Duc Thang University Ho Chi Minh City Vietnam; Department of Physics, Faculty of Science, University of Kurdistan 66177-15175 Sanandaj Iran; College of Electronic and Electrical Engineering, Sungkyunkwan University Suwon Korea mitragh@skku.edu; Institute of Research and Development, Duy Tan University Da Nang 550000 Vietnam

## Abstract

Correction for ‘The mechanical, electronic, optical and thermoelectric properties of two-dimensional honeycomb-like of XSb (X = Si, Ge, Sn) monolayers: a first-principles calculations’ by Asadollah Bafekry *et al.*, *RSC Adv.*, 2020, **10**, 30398–30405, DOI: 10.1039/D0RA05587E.

The authors regret that the name of one of the authors (Fazel Shojaei) was shown incorrectly in the original article. The corrected author list is as shown above.

The Royal Society of Chemistry apologises for these errors and any consequent inconvenience to authors and readers.

## Supplementary Material

