# Supplementary material for: The mechanical, electronic, optical and thermoelectric properties of two-dimensional honeycomb-like of XSb (X = Si, Ge, Sn) monolayers: a first-principles calculations
Source: RSC Adv. 2020 Aug 17;10(51):30398–405. doi: 10.1039/d0ra05587e (PMC9056272; doi:10.1039/d0ra05587e)
Supplement: RA-010-D0RA05587E-s001 [file RA-010-D0RA05587E-s001.pdf]

Electronic Supplementary Material (ESI) for RSC Advances.
This journal is © The Royal Society of Chemistry 2020

Supplementary Information

The mechanical, electroic, optical and thermoelectric properties of two-dimensional honeycomb-like of XSb (X=Si, Ge, Sn) monolayers: A first-principles calculations

A. Bafekry*[1,2], F. Shojaei[3], D. M. Hoat[4,5], M. Shahrokhi[6], M. Ghergherehchi[7], C. Nguyen[8]

[1]Department of Physics, University of Guilan, 41335-1914, Rasht, Iran

[2]Department of Physics, University of Antwerp, Groenenborgerlaan 171, B-2020 Antwerp, Belgium

[3]School of Nano Science, Institute for Research in Fundamental Sciences (IPM), 19395-5531 Tehran, Iran

[4]Computational Laboratory for Advanced Materials and Structures, Advanced Institute of Materials Science, Ton Duc Thang University, Ho Chi Minh City, Vietnam

[5]Faculty of Applied Sciences, Ton Duc Thang University, Ho Chi Minh City, Vietnam

[6]University Lyon, ENS de Lyon, CNRS, Universit Claude Bernard Lyon 1, Laboratoire de Chimie UMR 5182, F-69342 Lyon, France

[7]College of Electronic and Electrical Engineering, Sungkyun kwan University, Suwon, Korea

[8]Institute of Research and Development, Duy Tan University, Da Nang 550000, Viet-nam.

∗ Bafekry.asad@gmail.com

Computational details for optical properties calculations

In the random phase approximation, the imaginary part of the interband dielectric permittivity is given by [1]:

$$\mathrm{Im}\,\varepsilon_{\alpha\beta}(\omega) = \frac{4\pi^2 e^2}{\Omega} \lim_{q\to 0} \frac{1}{|q|^2} \sum_{c,v,k} 2 w_k \delta(\varepsilon_{ck} - \varepsilon_{vk} - \omega) \times \langle u_{ck+e_\alpha q} | u_{vk} \rangle \langle u_{ck+e_\beta q} | u_{vk} \rangle^* \quad (1)$$

where q is the Bloch vector of the incident wave, $w_k$is the **k**-point weight and the band indices c and v are restricted to the conduction and the valence band states, respectively. By using the $\mathrm{Im}\,\varepsilon_{\alpha\beta}(\omega)$, one can determine the corresponding real part via the Kramers–Kronig relations:

$$\mathrm{Re}\,\varepsilon_{\alpha\beta}(\omega) = 1 + \frac{2}{\pi} P \int_0^\infty \frac{\omega' \,\mathrm{Im}\,\varepsilon_{\alpha\beta}(\omega')}{(\omega')^2 - \omega^2 + i\eta} d\omega' \quad (2)$$

where P denotes the principle value and η is the complex shift.By taking into account the contribution of intraband transitions for metals [1], it is obtained that:

$$\mathrm{Im}\,\varepsilon_{\alpha\beta}^{[\mathrm{int}ra]}(\omega) = \frac{\Gamma \omega_{pl,\alpha\beta}^2}{\omega(\omega^2 + \Gamma^2)} \quad (3)$$

$$\mathrm{Re}\,\varepsilon_{\alpha\beta}^{[\mathrm{int}ra]}(\omega) = 1 - \frac{\omega_{pl,\alpha\beta}^2}{\omega(\omega^2 + \Gamma^2)} \quad (4)$$

In these equations ω_{pl} is the plasma frequency and Γ is the life time broadening:

$$\omega_{pl}^2 = \frac{n_e e^2}{\varepsilon_0 m^*} \quad (5)$$

where $n_e$is the number density of electrons, e is the electric charge, m^* is the effective mass of the electron.The adsorption coefficient determined as:

$$a_{\alpha\beta}(\omega) = \frac{2\omega k_{\alpha\beta}(\omega)}{c} \quad (6)$$

where $k_{\alpha\beta}$is imaginary part of the complex refractive index and c is the speed of light in vacuum, known as the extinction index. It is given by the following relations

$$k_{\alpha\beta}(\omega) = \sqrt{\frac{|\varepsilon_{\alpha\beta}(\omega) - \mathrm{Re}\,\varepsilon_{\alpha\beta}(\omega)|}{2}} \quad (7)$$

The reflectivity is given by

$$R_{ij}(\omega) = \frac{(n-1)^2 + k^2}{(n+1)^2 + k^2} \quad (8)$$

where *n* and *k* are real and imaginary parts of the complex refractive index, which are known as the refractive index and the extinction index, respectively.

References

[1] F. Wooten, **Optical Properties of Solids**, Academic press (2013).

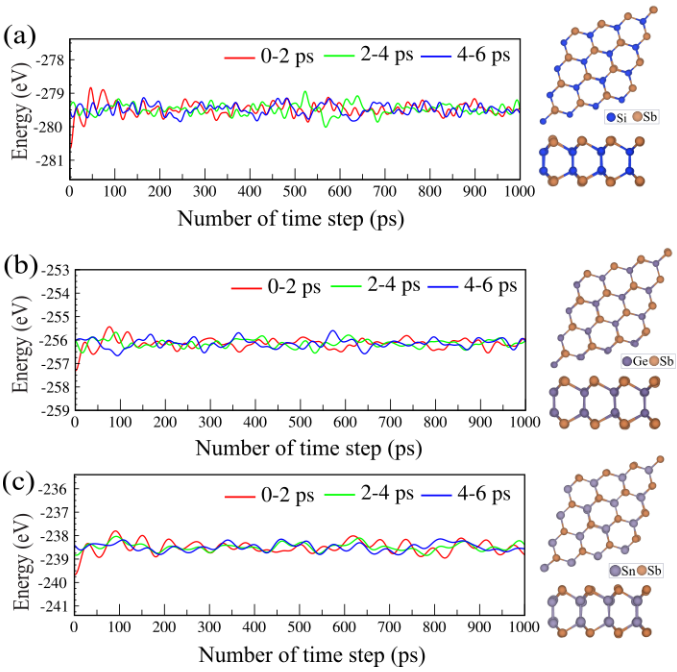

Fig. S1: AIMD simulation of (a) SiSb , (b) GeSb and (c) SnSb monolayers at 300 K. The optimized structures are indicated in the right of panel.
